# ATP release during cell swelling activates a Ca^2+^-dependent Cl^−^ current by autocrine mechanism in mouse hippocampal microglia

**DOI:** 10.1038/s41598-017-04452-8

**Published:** 2017-06-23

**Authors:** E. Murana, F. Pagani, B. Basilico, M. Sundukova, L. Batti, S. Di Angelantonio, B. Cortese, A. Grimaldi, A. Francioso, P. Heppenstall, P. Bregestovski, C. Limatola, D. Ragozzino

**Affiliations:** 1 Department of Physiology and Pharmacology, Sapienza University, Rome, Italy; 2 Istituto Italiano di Tecnologia, CLNS@Sapienza, Rome, Italy; 3 Mouse Biology Unit, EMBL, Monterotondo, Italy; 4 CNR NANOTEC - Istituto di Nanotecnologia, Department of Physics, University Sapienza, Rome, Italy; 5 Aix Marseille University, Inserm, INS, Institut de Neurosciences des Systèmes, Marseille, France; 6 IRCCS Neuromed, Via Atinese, Pozzilli, Italy; 7 Pasteur Institute – Department of Physiology and Pharmacology, Sapienza University, Rome, Italy; 8 Department of Biochemistry, “Sapienza” University of Rome, Rome, Italy

## Abstract

Microglia cells, resident immune cells of the brain, survey brain parenchyma by dynamically extending and retracting their processes. Cl^−^ channels, activated in the cellular response to stretch/swelling, take part in several functions deeply connected with microglia physiology, including cell shape changes, proliferation, differentiation and migration. However, the molecular identity and functional properties of these Cl^−^ channels are largely unknown. We investigated the properties of swelling-activated currents in microglial from acute hippocampal slices of *Cx3cr1* ^+/*GFP*^ mice by whole-cell patch-clamp and imaging techniques. The exposure of cells to a mild hypotonic medium, caused an outward rectifying current, developing in 5–10 minutes and reverting upon stimulus washout. This current, required for microglia ability to extend processes towards a damage signal, was carried mainly by Cl^−^ ions and dependent on intracellular Ca^2+^. Moreover, it involved swelling-induced ATP release. We identified a purine-dependent mechanism, likely constituting an amplification pathway of current activation: under hypotonic conditions, ATP release triggered the Ca^2+^-dependent activation of anionic channels by autocrine purine receptors stimulation. Our study on native microglia describes for the first time the functional properties of stretch/swelling-activated currents, representing a key element in microglia ability to monitor the brain parenchyma.

## Introduction

Microglia are resident macrophages surveying central nervous system parenchyma. They are traditionally linked to immune responses and inflammatory diseases, as they respond to CNS injury by changing morphology, migrating to the site of damage and becoming phagocytic^1, 2^. In addition, through their continuous scanning of extracellular space, microglia carry on fundamental functions in the healthy brain, influencing neuronal activity and synaptic connections^3^.

Although considered electrically silent, microglia express different patterns of ionic channels depending on the physiological context^4^, which change upon tissue challenge^5^. In particular, microglia express volume/swelling-activated anionic channels^6^, involved in the process of regulatory volume decrease^7^ and possibly implicated in other cellular functions, including proliferation^8^, phagocytosis^7^ and resting membrane potential setting^9^. Importantly, these channels are also involved in microglia role as brain caretaker, as they are fundamental in migration and processes rearrangement^10^, in lamellipodia formation during phagocytosis^7^ and in production of new cellular processes during transformation from amoeboid to ramified/resting form^11^.

Volume-regulated anion channels (VRAC) are ubiquitously expressed, playing a key role in cell volume regulation^6, 12^. As reported in several cell types, VRAC activate slowly upon extracellular hypotonic challenge, displaying outward rectification^6, 13^; in addition current can be activated by decrease in ionic strength or intracellular stimuli^14, 15^. Although extensively characterized by electrophysiology and pharmacology, the molecular identity of volume activated anionic channels is not yet fully clarified^12^. Recently, it has been proposed that LRRC8 is essential component of volume-regulated Cl^−^ channels, while several unrelated molecules have been previously involved, including bestrophins and TMEM16 proteins^16^. In addition, membrane stretch can result in the activation of pannexin hemichannels and maxi anion channels^17, 18^.

Importantly, volume regulated channels are permeable to organic anions^6^ and together with pannexins and maxi anion channels, are readily gated in response to hypotonic stress, constituting a preferential path for ATP efflux upon cell swelling^18^. Due to the role for ATP as paracrine and autocrine mediator, all the mechanisms by which intracellular nucleotides are exported to extracellular compartment deserve elucidation. This is particularly relevant in microglia, given the central role of ATP in microglia biology^19^ and the possibility of influencing neuronal activity through purine release. Aberrations in such functions are believed to underlie many disease states in the brain, as swell-activated anion channel can be involved in the release of glutamate after a stroke or trauma exacerbating excitotoxic damage and causing neuronal cell death^14, 20^. Thus, the relationship between changes in cell structure and chloride permeability could be relevant for microglia behaviour in physiological and pathological contexts.

Volume activated Cl^−^ current has been characterized in rat cultured microglia^7, 8, 14^ as well as in microglia cell lines^21^. However, although largely used, these reduced preparations cannot be considered as an exhaustive model of microglia as they cannot tell much about the modifications of microglia physiological properties, arising from tissue interactions^22^. Here, we report for the first time the expression of a volume activated current in microglia cells in acute murine brain slices. In addition, using a combination of patch-clamp technique and genetically encoded sensors for the analysis of changes in intracellular concentration of Cl^−^ and ATP in brain slices and cultured cell lines, we determined the physiological and pharmacological properties of swell-activated currents in microglia. We report that these currents are characterized by Cl^−^ selectivity, Ca^2+^- dependency and autocrine modulation by purines, highlighting the importance of volume activated ATP release as a potential signaling pathway triggered by microglia.

## Results

### Swelling-induced chloride currents activation in hippocampal microglia

**Figure 1:**
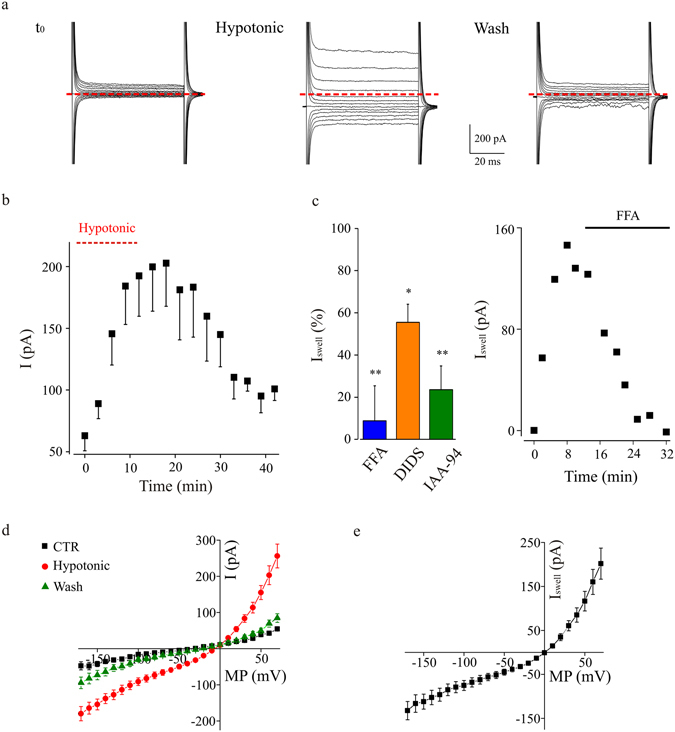
Induction of a swelling-activated current by hypotonic stimulation in microglia from acute hippocampal slices. (**a**) Whole-cell currents recorded from a microglia cell in acute hippocampal slice (HP = −70 mV; test potentials from −170 to +70 mV; 10 mV increments), chronically exposed to a hypotonic extracellular solution (8–10% dilution). Left, current traces recorded after the establishment of the whole cell configuration (t_0_); middle, currents recorded after 12 minutes of hypotonic stimulation (Hypotonic); right: currents traces recorded after 21 minutes of washout in normotonic solution (Wash). The zero current level is indicated by dotted red line. (**b**) Time course of swell-induced current recorded in hippocampal microglia cells (n = 6). Changes in amplitude (measured at +50 mV) are plotted against time after establishment of the whole-cell configuration. (**c**) Left: bar chart representing the reduction of swell-activated current caused by slices superfusion with flufenamic acid (FFA, 200 μM; n = 7; blue bar), diisothiocyanatostilbene-2,2′-disolfonic acid (DIDS, 150 μM; n = 4; orange bar), or indanyloxyacetic acid 94 (IAA-94, 500 μM; n = 5; green bar), respectively. *p < 0.05 and **p < 0.01 vs control, t-test. Right: representative time course of swell-activated current block by acute application of FFA. (**d**) Mean current-voltage relationships (n = 7) for the current recorded in control (black squares, CTR), after 18 minutes of acute hypotonic stimulation (red dots) and after washout (green triangles). (**e**) Average current-voltage relationship (n = 7) of the swell-activated current recorded in microglia cells from acute hippocampal slices under acute hypotonic stimulation. The current was isolated by subtraction of the current amplitudes measured prior to hypotonic stimulus from those at 18 minutes of hypotonic stimulation.

Membrane currents were recorded from microglia cells in CA1 stratum radiatum of acute hippocampal slices (P10–P25). When these cells, usually displaying a limited array of voltage dependent currents^23^, were exposed to a mild hypotonic medium (8–10% dilution), we observed the time-dependent activation of an outward rectifying current (Fig. 1A and B), which was absent when slices were maintained in isotonic conditions (Supplementary Fig. S1). The voltage dependence of the swelling-activated current (I_Swell_) was investigated by applying a series of voltage steps from a holding potential of −70 mV. The current, associated with a progressive increase in the leakage current at −70 mV (Fig. 1A), displayed a mild outward rectification, but not the time dependent inactivation at positive potentials, typical of swelling-induced currents^6^. The amplitude of I_Swell_ increased over time, reaching a plateau within 10–12 minutes (Fig. 1B) and disappeared upon stimulus washout, when slices were perfused with normotonic extracellular solution (n = 6; Fig. 1A and B; p < 0.005 after 15 min wash). Swelling-activated currents were inhibited by anion channel blockers. In particular, the acute application of flufenamic acid (FFA, 200 μM; n = 6, p < 0.05), indanyloxyacetic acid 94 (IAA-94, 500 μM; n = 5, p < 0.05) or diisothiocyanatostilbene-2,2′-disolfonic acid (DIDS, 150 μM; n = 4, p < 0.05) strongly reduced the amplitude of I_Swell_ (Fig. 1C). The reversal potential of I_Swell_ was accurately determined in a separate set of experiments, where hypotonic medium was applied acutely after few minutes of whole cell dialysis, to allow a complete equilibrium of intracellular solution (Fig. 1D and E). In these experiments, hypotonic stimulation activated in microglia cells the typical outward rectifying current, reverting at −0.2 ± 2.0 mV (n = 7; Fig. 1E), showing the typical time-dependent activation (not shown) and disappearing after washout to normotonic medium (Fig. 1D).

**Figure 2:**
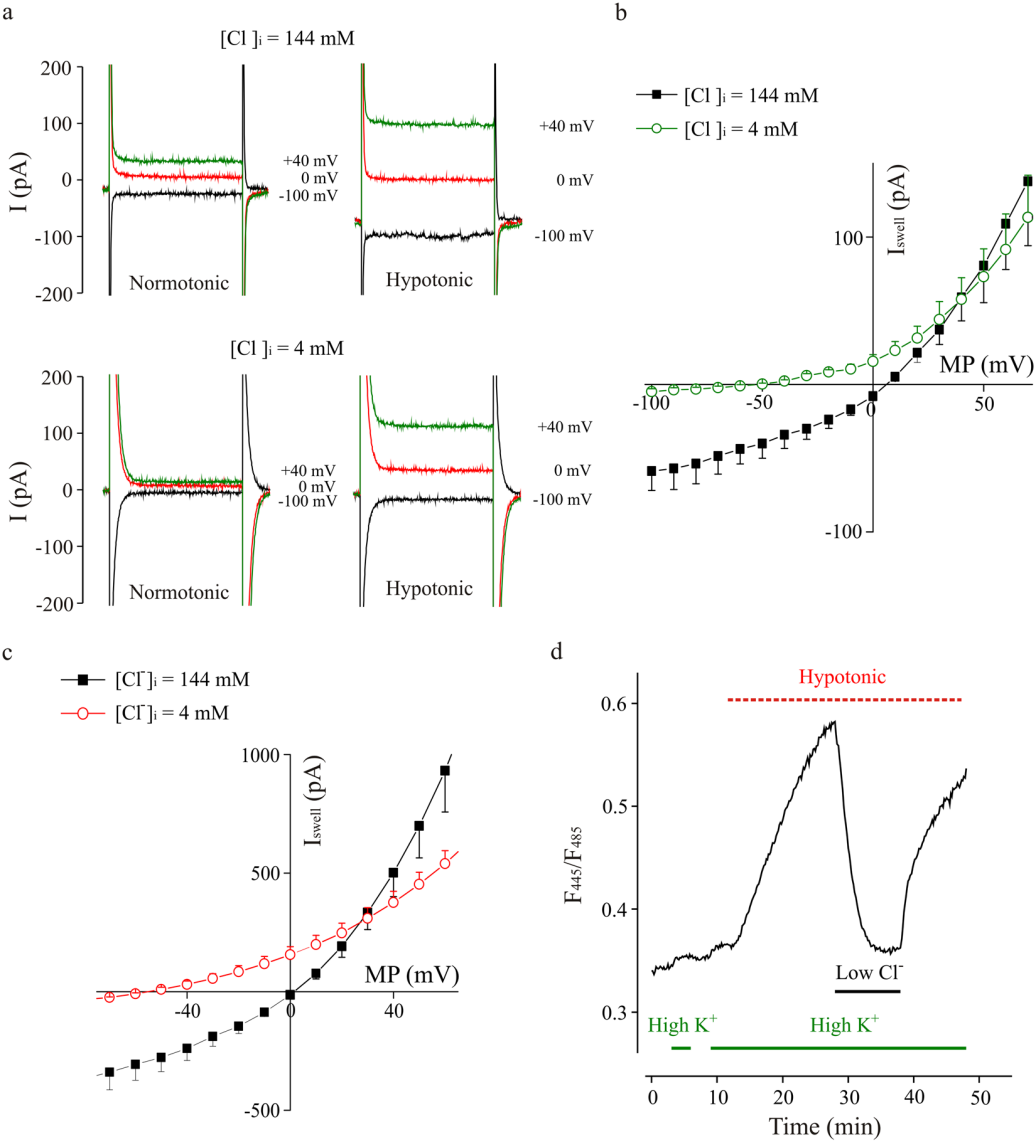
Hypotonic-activated current is driven by Cl^−^ ions, in microglia cells. (**a**) Representative whole-cell currents recorded under acute application of the hypotonic extracellular solution (8–10% dilution), at the indicated membrane potentials, with a standard ([Cl^−^]_i_ = 144 mM; upper traces) or a low chloride ([Cl^−^]_i_ = 4 mM; lower traces) pipette solution. As indicated, the left traces were recorded in normotonic condition, just before applying the hypotonic medium; the right traces were recorded 21 minutes after hypotonic stimulation. (**b**) Current-voltage relationships of the currents recorded in control condition ([Cl^−^]_i_ = 144 mM; black squares; n = 3) and with a low chloride pipette solution ([Cl^−^]_i_ = 4 mM; green circles; n = 4; HP = −70 mV) during acute application of hypotonic medium. Note the shift of the current reversal potential to more negative values. (**c**) Current-voltage relationships recorded in BV-2 cells, exposed acutely to a hypotonic medium (205 mOsm), in control condition ([Cl^−^]_i_ = 144 mM; black squares, n = 9) and lowering Cl^−^ concentration ([Cl^−^]_i_ = 4 mM; red circles; n = 7) in the pipette solution. Graph represents the swell-activated current (I_Swell_; subtraction of current in control to that under hypotonic stimulation) to eliminate the overlap with endogenous cell conductances. Note the shift of the current reversal potential to more negative values. (**d**) Time course of fluorescence ratio increase induced by the exposure of BV-2 cells, transfected with Cl^−^ Sensor, to a hypotonic solution (200 mOsm, red bar) in normal or low extracellular Cl^−^ (6 mM, black bar). Cells are concomitantly depolarized by high K^+^ extracellular medium (60 mM, green bar), which *per se* did not cause significant changes in the fluorescence ratio.

To establish the ion selectivity of the swelling-activated current, intracellular Cl^−^ concentration was decreased to 4 mM by substitution with gluconate (Fig. 2a and b). The use of K-gluconate based pipette solution caused a shift of the current reversal potential to more negative values (E_rev_ = −51.0 ± 7.9 mV with K-gluconate; p < 0.01; n = 4 Fig. 2b). Recordings performed with a N-methyl-D-glucamine based intracellular solution did not show a depolarizing shift of I_Swell_ reversal potential (E_rev_ = −7.8 ± 0.8 mV; n = 4; p < 0.001; data not shown), as expected in case of a relevant cationic component. These observations indicate that swelling-activated current are mainly based on Cl^−^ permeability.

To directly monitor the Cl^−^ flux caused by the activation of swelling activated currents, a set of experiments was performed in cultured BV-2 cells transfected with a fluorescent Cl^−^ Sensor, a protein indicator that allows a ratiometric and not invasive analysis of intracellular chloride concentration^24, 25^. When exposed to a hypotonic stimulus, BV-2 cells displayed a swelling induced current, showing the typical outward rectification (Supplementary Fig. S2), Cl^−^ permeability (Fig. 2c) and sensitivity to FFA (Supplementary Fig. S2). To visualize Cl^−^ flux, Cl-Sensor expressing BV-2 cells were exposed to a hypotonic extracellular medium designed to simultaneously activate I_Swell_ and depolarize membrane potential (Hypotonic/High K^+^ solution), in order to change the electrochemical gradient for Cl^−^ and favor its entry into the cells. In this condition, we observed an increase in the fluorescence ratio, indicating Cl^−^ influx, which was reverted upon shifting to a low Cl^−^ (6 mM) extracellular solution (Fig. 2d). These data indicate that in BV-2 microglia cells, hypotonic stimulation activates a membrane conductance carried by Cl^−^ ions, whose flux is driven by electrochemical Cl^−^ gradient.

Together, these results demonstrate that hypotonic stimulus induces in microglia cells the activation of membrane currents through Cl^−^ permeable channels.

### Swelling-activated currents require microglia ATP release

**Figure 3:**
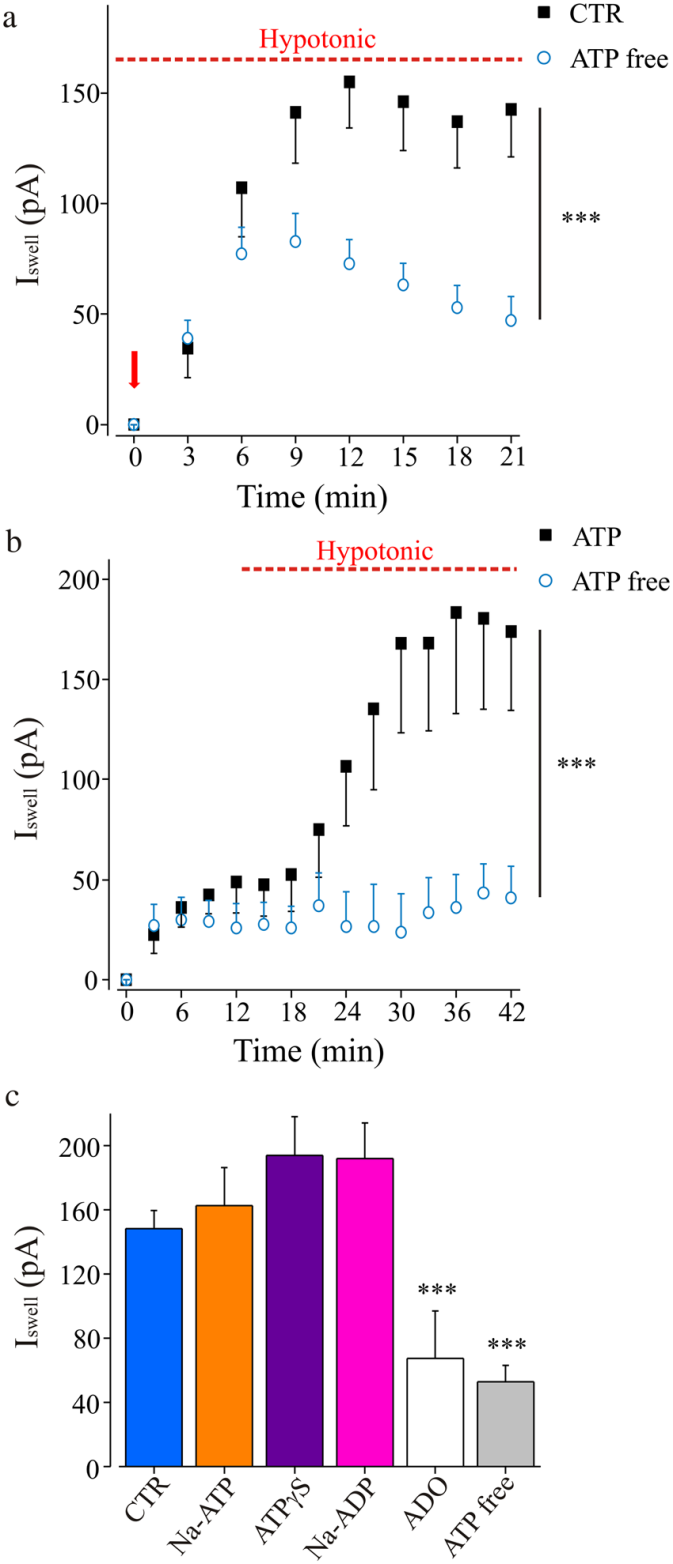
Microglia swelling currents activation requires intracellular purines. (**a**) Time course of I_swell_ recorded with control pipette solution (2 mM Mg-ATP, n = 18; black) or with an ATP-free solution (n = 27; cyan; ***p = 0.001, two-way ANOVA, Holm-Sidak) under chronic hypotonic stimulation. **(b)** Time course of the swell-activated current evoked by acute application of hypotonic stimulus, in the presence of 2 mM Mg-ATP (black, n = 5) or without Mg-ATP (cyan, n = 6) in the intracellular solution. ***p = 0.001, two-way ANOVA, Holm-Sidak. Notice that after complete intracellular dialysis, I_Swell_ is absent when ATP was omitted from pipette solution. (**c**) Bar chart representing swell-activated current amplitude (18 minutes after hypotonic stimulation; MP =  + 50 mV) recorded with substitutions of Mg-ATP (2 mM) in the pipette solution. Each experimental set was compared with respective internal controls (recorded in the same experimental days with Mg-ATP in the pipette solution). CTR bar represents mean I_Swell_ amplitude of all internal controls (2 mM Mg-ATP in the pipette solution, n = 48; blue bar). Na-ATP (2 mM, orange; n = 7 vs n = 10; p > 0.05, t-test), ATPγS (2 mM, violet; n = 11 vs n = 6; p > 0.05, t-test), Na-ADP (2 mM, pink; n = 12 vs n = 15; p > 0.05, t-test), adenosine (ADO, 2 mM, white; n = 10 vs n = 18; ***, p < 0.001, t-test) or with an ATP-free solution (gray; n = 23 vs n = 17; ***, p < 0.001, t-test).

Swelling activated anionic channels are present in a large variety of cells, showing different mechanisms of activation and control. In particular, intracellular ATP is known to be necessary for the activation of volume-regulated anionic currents^11, 14^. Thus, to determine whether ATP is required for the activation of I_Swell_, we exposed hippocampal microglia to hypotonic stimulus in the absence of ATP in the intracellular solution. As shown in Fig. 3a, in this condition, the amplitude of I_Swell_ was strongly reduced. However, hypotonic challenge induced the transient activation of an outward rectifying current, showing similar I/V relation (not shown), possibly due to the incomplete dialysis of intracellular ATP. Consistently, when microglia cells were acutely treated with hypotonic medium after intracellular dialysis, I_Swell_ was completely abolished (n = 6; Fig. 3b).

ATP is the major donor of phosphoric group during phosphorylation processes, thus the decrease of swelling-induced current in the absence of ATP might result from “run-down” processes^26^. To investigate if ATP-dependent phosphorylation processes were required for I_Swell_ activation, membrane currents were recorded with a pipette solution in which Mg-ATP was substituted by Na-ATP, unable to participate to classical reactions of phosphate group transfer^27^. In these conditions of disfavoring phosphorylations, hypotonic stimulus induced a swell-activated current similar to control (n = 9; p = 0.6; Fig. 3c). Similar results were obtained when intracellular ATP was substituted by ATPγS (2 mM), a slowly hydrolyzable ATP analog which prevents dephosphorylation, due to the formation of irreversibly thiophosphorylated residues (n = 21; p > 0.16; Fig. 3c). Remarkably, ATP could be also replaced by ADP. Indeed, in presence of ADP, I_Swell_ occurred showing typical amplitude (n = 13; Fig. 3c), while when the pipette solution contained adenosine (ADO, n = 10; Fig. 3c), the current failed to be activated. Together, these results show that intracellular purines are required for I_Swell_ activation, but their role is likely not linked to phosphorylation or ATPase reactions.

It is known that brain cells can extrude ATP or ADP in different ways including pannexin hemichannels^17, 28–30^. Thus, we hypothesized that in our experimental conditions, ATP could be released by microglia cells, favoring I_Swell_ activation by triggering a purinergic pathway.

**Figure 4:**
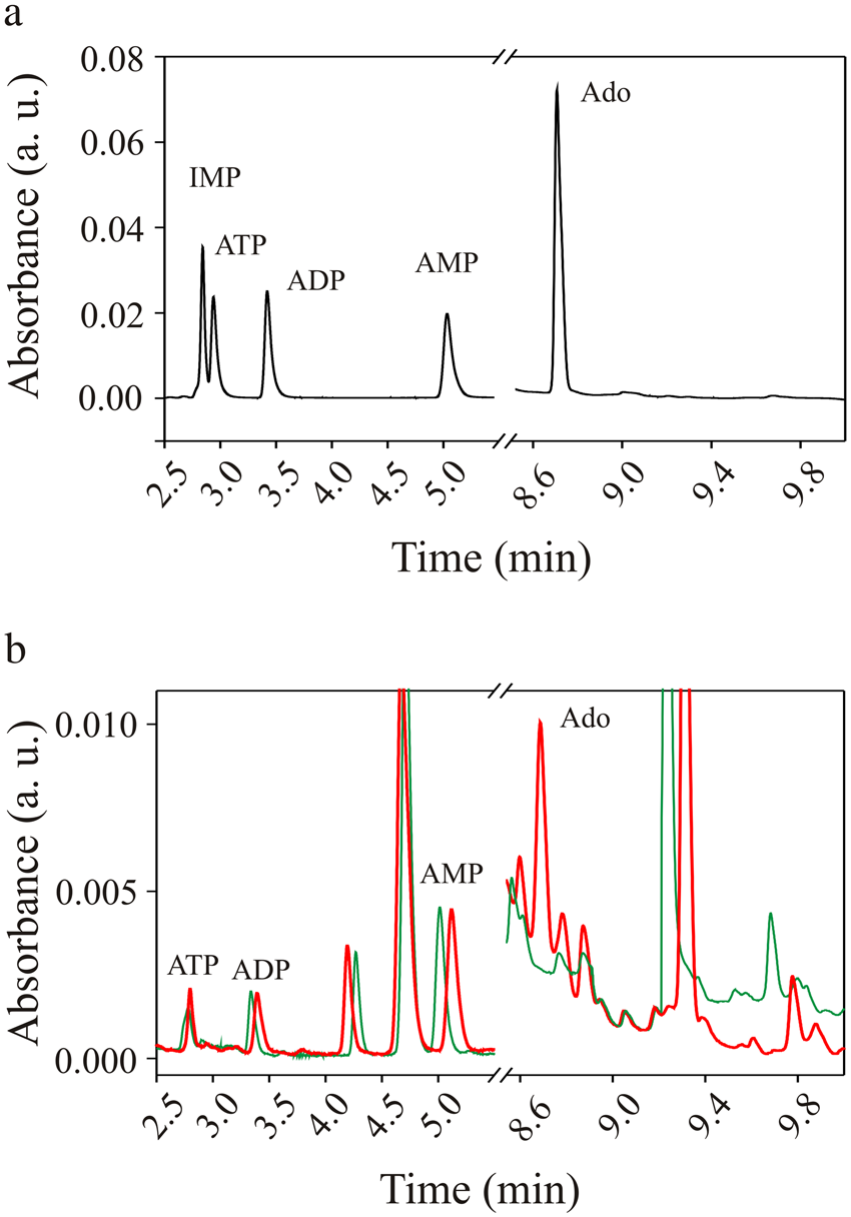
Chromatographic analyses of microglia medium. (**a**) Representative chromatogram of nucleotides standards. (**b**) Chromatogram of samples collected from microglia cultures following treatment in normotonic (green) and hypotonic (red) condition for 5 minutes.

To verify this hypothesis we, first, ascertained purine release in primary cultured microglia under hypotonic conditions. On this purpose, we collected cell culture medium after hypotonic challenge and analyzed it by ultraperformance liquid chromatography. When microglia cultures were exposed for 5 minutes to hypotonic stimulus, the culture medium contained a submicromolar concentration of adenosine, which was not observed in unstimulated cultures (Fig. 4). Concentrations of ATP, ADP and AMP were unchanged by hypotonic stimulation (Fig. 4). These results show that microglia cells are able to release purines when exposed to swelling conditions.

**Figure 5:**
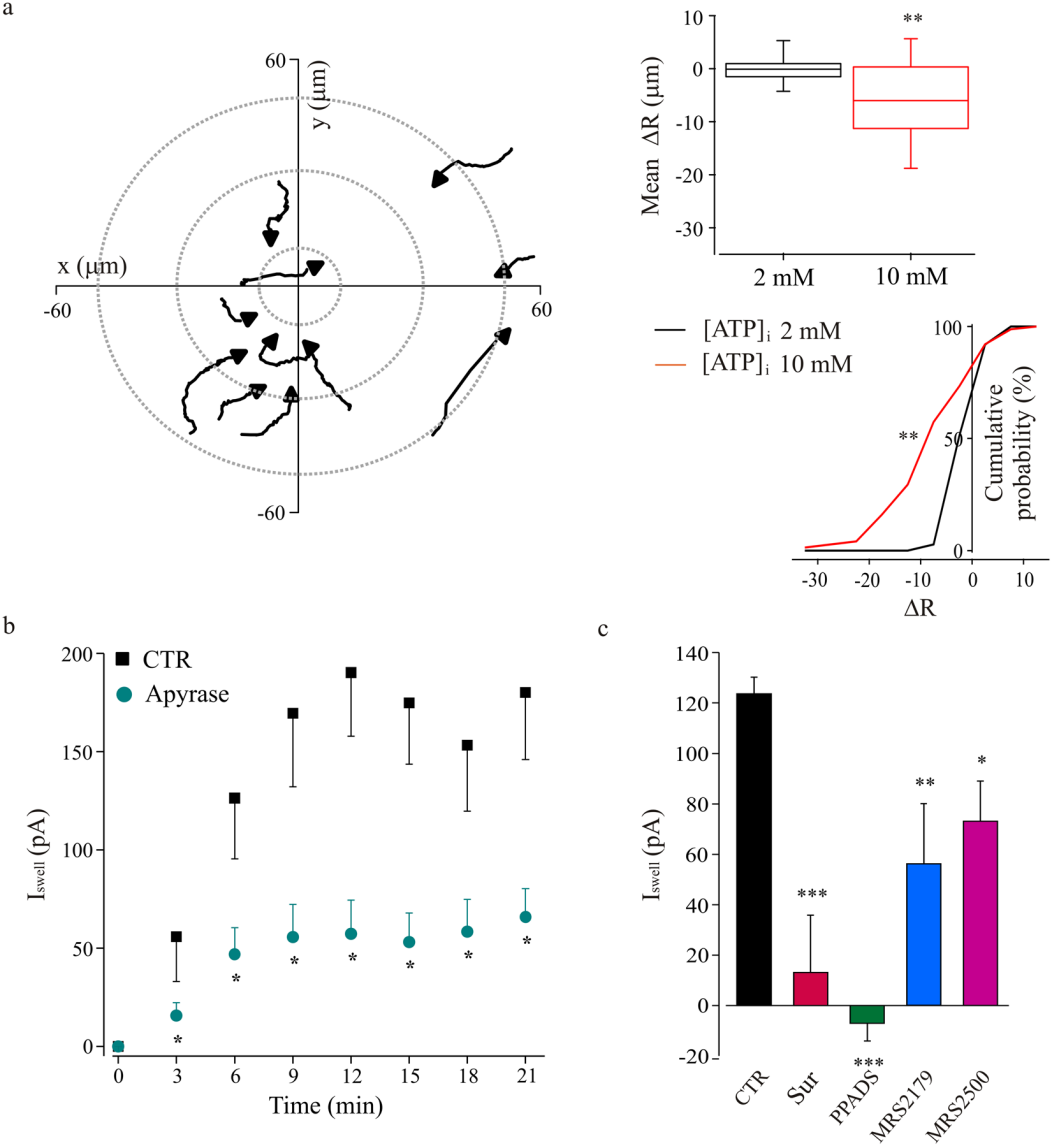
Microglia swelling currents activation requires purines release and P2R activation. (**a**) Microglia processes recruitment after I_Swell_ activation in patch clamped microglia in acute hippocampal slice. Left: tracking of microglia processes movement in respect to the recorded cell, dialyzed with 10 mM ATP containing pipette solution, during hypotonic stimulation. Graph origin corresponds to the recorded cell (x = 0, y = 0); gray dotted lines define concentric areas with increasing radial distance. Each trace corresponds to a single process, with black arrows indicating the movement direction. Right, upper panel: bar chart representing the mean displacement of microglia processes during extracellular hypotonic stimulation, evaluated in respect to cells recorded with a pipette solution containing 2 mM (black box; ΔR = 0.005 ± 0.477 μm, n = 37 processes) or 10 mM (red box; ΔR = −6.31 ± 0.97 μm, n = 75) of ATP. **p < 0.01, t-test. Right, lower panel: cumulative distributions of microglia processes ΔR, under extracellular hypotonic stimulation, with a pipette solution containing 2 mM (black line) or 10 mM (red line) ATP. **p < 0.01, Kolmogorov-Smirnov test. (**b**) Time course of swell-activated currents evoked with the chronic hypotonic protocol, and recorded in control condition (n = 9; black squares) and in slices treated with apyrase (20 U/ml; n = 11; cyan dots; *p < 0.05, t-test). (**c**) Effects of purinergic receptors blockers on the swell-activated current amplitude. Bar char represents the amplitude of currents recorded in control condition (black, n = 18), in presence of suramine (500 μM, red, n = 4), PPADS (100 μM, green, n = 4), and two different blockers of P2Y1 receptor, MRS-2179 (100 μM, blue, n = 5) and MRS-2500 (1 μM, magenta, n = 10). I_Swell_ was evaluated after 12 minutes of hypotonic stimulation (MP =  + 50 mV). *p < 0.1, **p < 0.01 and ***p < 0.001 vs control; one-way ANOVA, Holm-Sidak.

To demonstrate that microglia are able to release ATP in our experimental conditions, we took advantage of their ability to rearrange processes toward a source of ATP^31^ (see also the legend to Supplementary Fig. S4). On this purpose, we analyzed the rearrangement of GFP-positive microglia processes around a recorded microglia cell under hypotonic stimulation (Fig. 5a), by tracking the movement of single processes^23^. Reconstructed processes trajectories showed that during I_Swell_ activation, microglia processes move in the direction of the recorded cell under hypotonic stimulation, in an ATP concentration-dependent manner. Indeed, the mean displacement of surrounding processes indicated a rearrangement towards the swelled cell when microglia cells were dialyzed with a pipette solution containing 10 mM ATP and a null net movement in presence of 2 mM ATP (p < 0.001, t-test; Fig. 5a). Conversely, in normotonic conditions, increasing intracellular ATP was ineffective in attracting microglia processes towards the recorded cell (not shown).

Consistently, we observed that when slices were treated with apyrase, an enzyme that degrades ATP and ADP to AMP (20 U/ml, 1 hour of pretreatment and application during recordings), hypotonic stimulation was unable to elicit the typical current response (n = 11, p < 0.05 vs CTR n = 9; Fig. 5b). In addition, we observed that purinergic receptors blockers suramin (500 μM, n = 4, p < 0.001) and PPADS (100 μM, n = 4, p < 0.001) prevented swell-induced current activation (Fig. 5c). Similarly, I_Swell_ showed a reduced amplitude in slices treated with selective antagonists of P2Y1 (MRS-2179: 100 μM, n = 5, p < 0.001; MRS-2500: 1 μM, n = 10, p < 0.01) receptor (Fig. 5c).

Altogether, these data show that ATP is necessary for current activation and is released during I_Swell_ activation raising the possibility of an extracellular site of action through purininergic receptors.

### Involvement of hemichannels in microglia ATP release

**Figure 6:**
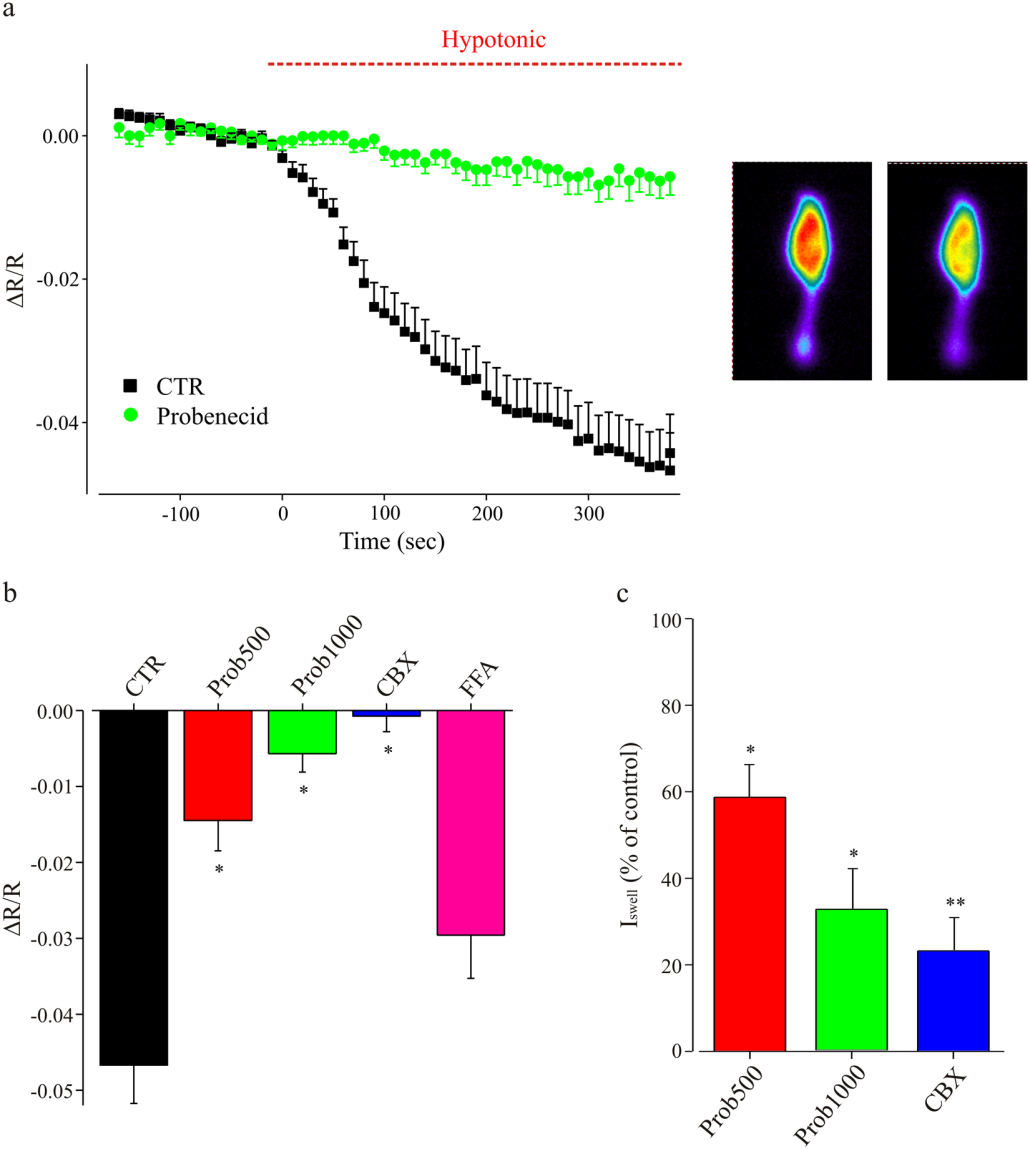
Cell swelling induces ATP release from microglia cells. (**a**) Left, time course of the fluorescence ratio obtained by the acute exposure of BV-2 cells transfected with a FRET-based ATP sensor, to an hypotonic extracellular solution (205 mOsm, red bar) in absence (black; n = 31) or in presence of probenecid (1 mM, green; n = 16; p < 0.05 at 5 minutes of hypotonic medium application); right, representative fluorescence images of a transfected BV2 cell, in normotonic condition (left) and under hypotonic stimulation (right); notice the decrease in fluorescence ratio, indicating a reduction in [ATP]_i_. (**b**) Bar chart representing the fluorescence ratio evaluated in ATP-sensor transfected BV-2 cells, in response to hypotonic medium (200 mOsm; 5 minutes of application) in the absence of blockers (CTR, black, n = 31) or in presence of probenecid (500 μM, red, n = 24; 1 mM, green, n = 16) or CBX (100 μM, blue, n = 13) or FFA (pink, n = 31). *p < 0.05 vs CTR, one-way ANOVA, Holm-Sidak. (**c**) Bar chart showing I_Swell_ in slices treated with probenecid (500 μM or 1 mM) or CBX (100 μM), expressed as percentage of the current amplitude in internal controls (recorded in the same experimental days in untreated slices). Probenecid 500 μM: red; n = 13 vs n = 5; **p < 0.01, t-test. Probenecid 1 mM: green, n = 9 vs n = 5; **p < 0.01, t-test. CBX: blue; n = 6 vs n = 4; **p < 0.01, t-test. I_Swell_ amplitude as in 5c. Statistics were performed on raw amplitude values.

To investigate the mechanisms of ATP release from microglia during hypotonic stimulation, we used transfected BV2 cells expressing a FRET based ATP sensor (Fig. 6a). In this reduced system, we monitored the dynamics of ATP levels in BV2 cells during the application of an acute hypotonic stimulus. Figure 6a shows sequential images of the ratio of CFP and YFP channels at monitoring of ATP sensor fluorescence. Hypotonic stimulus induced a significant decrease in the YFP/CFP emission ratio, indicating a decrease of intracellular [ATP] in microglia cells (Fig. 6a). To verify if the observed decrease in cell fluorescence was due to a release of ATP during hypotonic challenge, FRET experiments were performed in presence of pannexin hemichannel blockers CBX^32^ (100 μM) or probenecid^33^ (500 μM and 1 mM). The application of these blockers prevented the hypotonic-induced decrease of cell fluorescence, while that of FFA (200 μM mM) was ineffective, indicating that the decrement of intracellular [ATP] was associated to ATP release through hemichannels (Fig. 6a and b). A similar decrease in fluorescence ratio was observed in primary microglia cultures trasnsfected with ATP sensor (n = 5; not shown). These results suggest that pannexin1 hemichannels may be involved in ATP release during hypotonic stimulation^34, 35^.

Since ATP removal prevented I_Swell_ activation and hemichannels are associated to microglia purine release in hypotonic conditions, we used pannexin blockers to ascertain whether the activity of these conduits could interfere with hypotonic-induced current. When experiments were performed in the presence of carbenoxolone (CBX, 100 μM), I_Swell_ amplitude was strongly reduced (n = 7; Fig. 6c). Consistently, recordings performed in presence of the selective pannexin antagonist, probenecid (500 μM and 1 mM), showed a dose-dependent reduction in current amplitude (Fig. 6c), supporting the view of hemichannels involvement in swell-induced current activation. On the other hand, gadolinium (500 μM) a blocker of maxi anion channels was ineffective (n = 3; p = 0.4; not shown).

### Microglia swelling-activated current is [Ca^2+^]_i_ -dependent

Among the transduction pathways controlled by purine *P2Y* receptors activation, we hypothesized that ATP might promote the activation of I_Swell_ by inducing intracellular Ca^2+^ increase in microglia cells. Indeed Ca^2+^-activated anionic channels, present in a large variety of cells, are involved in microglia response to cell swelling^36^.

**Figure 7:**
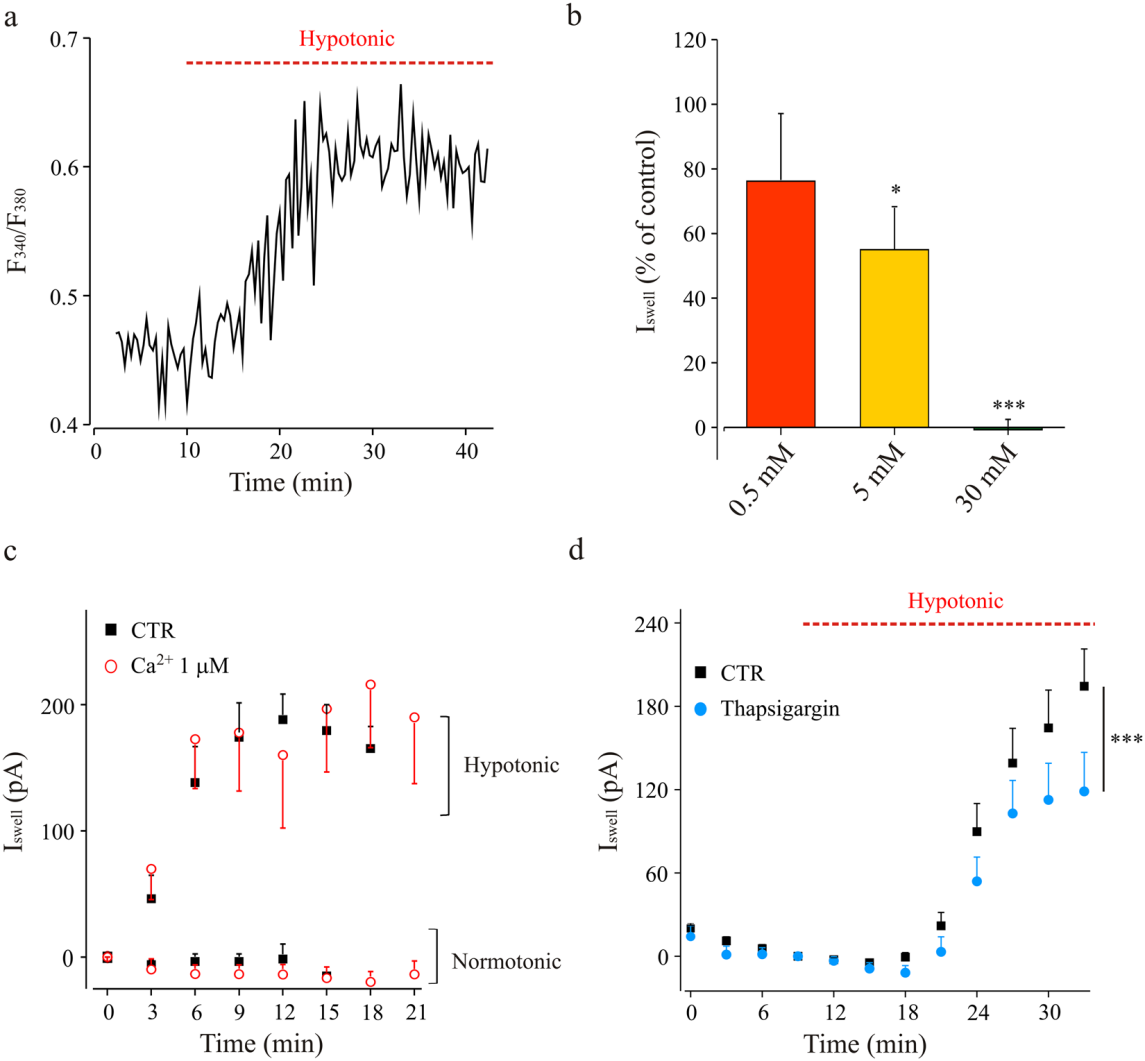
Intracellular Ca^2+^ increase is necessary for the hypotonic-induced current activation. (**a**) Representative trace of intracellular Ca^2+^ increase in a Fura-2-loaded (100 μM) microglia cell, recorded in hippocampal slices under acute hypotonic stimulation (red bar, 8–10% dilution). (**b**) Bar chart of I_Swell_ amplitude measured in presence of BAPTA 0.5, 5 and 30 mM in the pipette solution, expressed in percentage (see 6c). Internal controls were recorded in the same experimental days with EGTA 0.5 mM in the pipette solution. BAPTA 0.5 mM: red; n = 9 vs n = 9; p = 0.4, t-test. BAPTA 5 mM: yellow; n = 14 vs n = 25; p < 0.05, t-test. BAPTA 30 mM: green; n = 6 vs n = 8; p < 0.001, t-test. I_swell_ amplitude as in 5c. Statistics as in 6c. **(c)** Time-course of I_Swell_ amplitude recorded in control conditions (black squares, CTR) and with 1 μM free [Ca^2+^]_i_, (red circles) in the pipette solution, under normotonic or hypotonic extracellular stimulation, as indicated. Notice that, high [Ca^2+^]_i_ was unable to activate I_Swell_ (red circles, n = 6; p = 0.3 vs CTR, black squares, n = 8) in the absence of hypotonic stimulation, or to modulate it under hypotonic stimulation (red circles, n = 5; p = 0.5 vs control, black squares, n = 11). (**d**) Time course of I_Swell_ recorded with control pipette solution (n = 9; black) or containing thapsigargin (1 μM; n = 13; blue). ***p = 0.001, two-way ANOVA, Holm-Sidak.

Combined recordings of membrane currents and intracellular Ca^2+^ in Fura2 loaded hippocampal microglia cells in slices, indicated that intracellular Ca^2+^ increased during hypotonic stimulation, as shown by the increase in fluorescence ratio F_340_/F_380_ (from 0.40 ± 0.03 to 0.50 ± 0.06 after 20 minutes of hypotonic stimulus; p = 0.03; Fig. 7a). Then, to highlight the role of Ca^2+^ in the mechanisms of activation of swell-induced current, we changed the Ca^2+^ buffering capacity of the pipette solution. On this purpose, EGTA was substituted by BAPTA (0.5, 5 e 30 mM). With BAPTA 0.5 mM, swell-activated current was similar to that recorded in control condition; while, at increasing BAPTA concentrations, I_Swell_ amplitude was significantly reduced (5 mM), or failed to be activated by the hypoosmotic stimulus (30 mM; Fig. 7b). These data demonstrate that the activation of swelling current requires at least minimal increase in [Ca^2+^]_i_, which is avoided by the fast chelator BAPTA in the intracellular solution. However, [Ca^2+^]_i_ manipulation was not sufficient for I_Swell_ activation in the absence of hypotonic stimulation (Fig. 7c). Indeed, in microglia cells recorded in presence of high intracellular Ca^2+^ (1 µM), we did not observe the time dependent activation of the typical outward rectifying current, when kept in normotonic conditions (Fig. 7c), indicating that the sole presence of high intracellular Ca^2+^ is unable to elicit I_Swell_. Consistently, in the presence of high intracellular Ca^2+^, hypotonic challenge elicited a current response, which was indistinguishable from control (Fig. 7c). Thus, the swelling current is Ca^2+^-dependent but Ca^2+^ increase is not sufficient to activate it, likely constituting an amplificatory pathway or a *precondition*. To determine whether the source of Ca^2+^ involved in current activation was intracellular, we performed experiments with a pipette solution containing thapsigargin (1 μM), in order to deplete intracellular Ca^2+^ stores of the recorded microglia cell, limiting the effects on surrounding cells. To allow stores depletion, in these experiments we applied the hypotonic stimulus acutely, after 9 minutes of intracellular dialysis. In these experimental conditions, microglia recorded with thapsigargin showed reduced I_Swell_ amplitude in respect to controls (Fig. 7d). On the other hand, when we exposed slices to hypotonic challenge in the nominal absence of external Ca^2+^, I_Swell_ displayed the typical activation (data not shown; n = 5; p > 0.1 vs internal controls, n = 5; two-way ANOVA).

**Figure 8:**
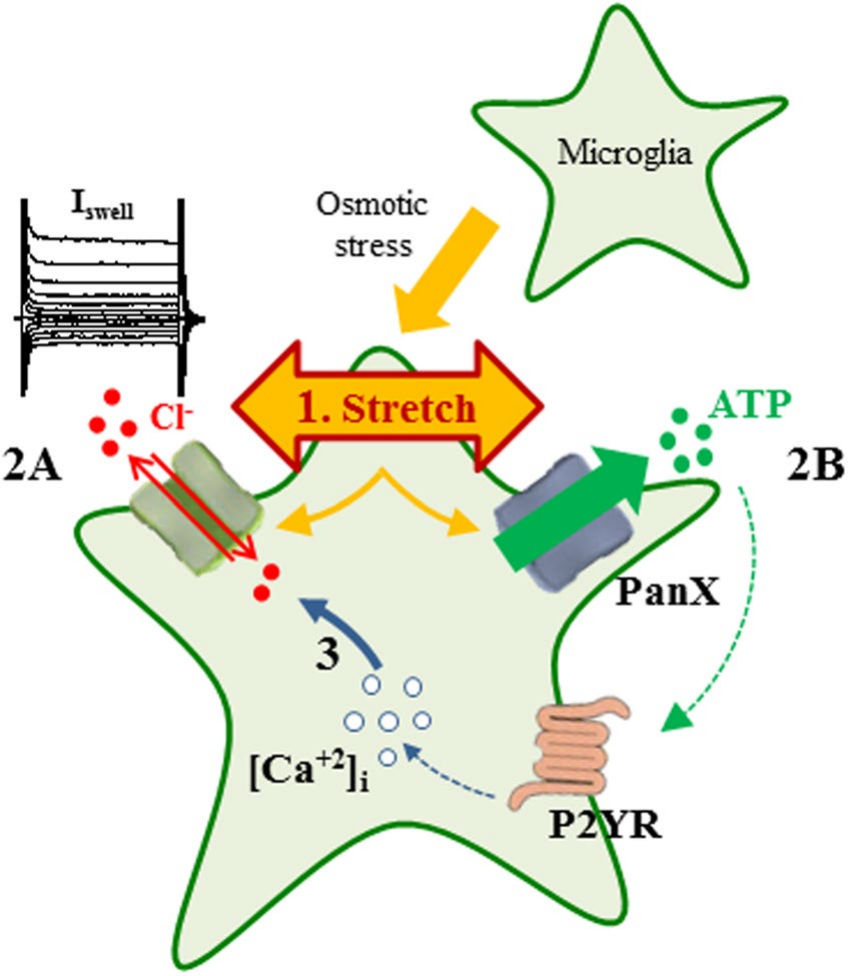
Model of the Ca^2+^ and ATP-dependent activation loop for hypotonic-induced currents in microglial cells. Osmotic stress causes cell swelling and a consequent membrane stretch (1), inducing a swell-dependent chloride current (**2A**) and ATP release through pannexin hemichannels (**2B**). ATP amplifies the current activation by binding to P2Y purinergic receptors (**P2YR**), possibly through Ca^2+^-dependent mechanisms. **I**_**Swell**_: swell-activated anionic channels; **PanX** pannexin1 hemichannels.

All together, these results indicate that ATP is released by microglia under hypotonic conditions through pannexin hemichannel and favors the Ca^2+^-dependent mechanisms of I_Swell_ activation acting on purinergic receptors (Fig. 8).

## Discussion

We report that hypotonic stimulation on microglia in acute hippocampal slices induces an ATP- and Ca^2+^-dependent chloride current. Membrane swell is also able to cause pannexin hemichannels-mediated ATP release that could amplify current activation through purinergic receptors and an increase of intracellular Ca^2+^ (Fig. 8). Swelling-released ATP may constitute a signal for microglia process recruitment. Our results show that microglia in acute hippocampal slices of *Cx3cr1* ^+/*GFP*^ mice display an outward rectifying current activated by hypotonic stimulation, similar to the current previously observed in microglia cultures^11, 14^. To activate the swelling current in microglia cells, we stimulated slices chronically or acutely with a mild hypo-osmotic stimulus, establishing a slight osmolarity delta between intracellular and extracellular sides. Our conditions, avoiding the use of very strong hypotonic stimuli^7, 14^, are suitable for the study of microglia currents activated by membrane swelling in slices. Several facts support the notion that I_Swell_ is specifically due to the induction of an osmotic delta across microglia membrane, disfavoring the possibility of chemical stimulation due to unknown substances released from other cells in the slice^37^. Indeed, (i) in the chronic protocol, all the cells in the slices are allowed to rebalance before starting the recording; (ii) I_Swell_ slowly activates after whole cell break through due to the transmembrane osmotic delta and (iii) disappears on return to normotonic condition; (iv) it can be activated also by hypertonic intracellular solution, while (v) it fails to develop when intracellular and extracellular solutions have similar osmolarity (Supplementary Fig. S1). Finally, I_Swell_ could be activated in several cells sequentially recorded in the same slice, which was always kept in the same medium.

In line with prevalent Cl^−^ selectivity, the reversal potential of the swell-activated currents was close to 0 mV in solutions with symmetrical Cl^−^ concentrations. The positive deviation observed in chronic experiments is likely due to the progressive dialysis with Cl-based solution during whole cell recording. Indeed, in experiment performed in acute conditions, after intracellular equilibration, I_Swell_ reversal potential was coincident with GHK equation predictions. In addition, upon equimolar substitution of Cl^−^ with gluconate in the intracellular solution, the expected negative shift in reversal potential was observed^11^. The deviation from predictions could be explained considering a significant permeability to gluconate, as reported by Gilbertson^38^ in rat taste neurons (gluconate/Cl permeability ratio 0.2). In alternative, it could be considered the possible permeability to other physiologically relevant anions, including ATP^7, 8, 14, 39^. It should be noted that the amplitude of I_Swell_ was reduced in Cl^−^ substituted solutions, suggesting sensitivity to the concentration of Cl^−^ ions^8, 11^.

Cl^−^ selectivity was confirmed by imaging experiments in BV2 cells transfected with Cl-sensor^24, 25^, highlighting Cl^−^ fluxes dependent on [Cl^−^]_e_. In these experiments, BV2 cells were concomitantly depolarized to increase Cl^−^ driving force under hypotonic stimulation.

Swelling-activated Cl^−^ current had a dependence on intracellular ATP, as the current showed only a transient activation in the absence of ATP in the intracellular solution. Remarkably, a striking ATP dependency was observed after intracellular dialysis, when the application of an acute hypotonic stimulus was ineffective in the absence of intracellular ATP. The role of intracellular ATP in the regulation of chloride currents activated by mechanical membrane deformation has been already highlighted in microglia and other cell types and is generally described in terms of an ATP-dependent current *run down* ^40^. However, ATP effect is apparently not dependent on ATP hydrolysis^11, 14, 40–42^. Differently from what reported in rat carotid body cells^43^, in our conditions, intracellular Mg-ATP could be substituted by analogs, like Na-ATP, ATPɣS or ADP, impairing phosphate transfer to substrate. This indicates that ATP is not necessary as P_i_-donor as in phosphorylation-based regulation mechanisms^27^. Conversly, we hypothesize that ATP is released by microglia cells during swelling, contributing to I_Swell_ activation in autocrine fashion^39, 42, 44^ (Fig. 8), as proposed for the mechanisms leading to cell volume regulation in different cellular systems^45^ and immune cells activation^46^.This conclusion is based on the absence of I_Swell_ when slices are treated with apyrase, an enzyme that degrades ATP and ADP to AMP and free inorganic phosphate^47^.

The effects of ATP removal by apyrase support the conclusion that ATP (or ADP) binds to one or more extracellular sites, stimulating anionic channels. Consistently, the broad-spectrum purinergic receptor antagonists, suramin and PPADS, abolished I_Swell_, suggesting the involvement of purinergic signaling in current activation. It should be noted that these compounds, although currently used, are not selective and even potentially targeting volume-activated channels^35, 48, 49^. In particular, the effect of PPADS and suramin could also be ascribed to their inhibition of ectoATPases^50, 51^, potentially impairing ATP conversion to ADP and adenosine. The involvement of purinergic signaling is further supported by the inhibitory action of selective antagonists for P2Y1 receptor. Indeed, P2YRs could be activated by ATP released during cell swelling and favor I_Swell_ activation by Ca^2+^-dependent mechanisms. Together, our data suggest that purinergic receptors are not required for I_Swell_ activation, but participate to an amplificatory pathway (Fig. 8), as the effects exerted by P2YRs antagonists are only partial. However, it cannot be excluded that a specific extracellular purine-binding site is functionally associated to anionic channels activation.

A critical issue is the mechanism by which ATP is released by microglia cells. The release of ATP through volume regulated channels has been proposed long time ago in hepatoma^39^ and endothelial cells^52^ and more recently in astrocytes and Raw macrophages^44, 53, 54^. Our conclusion that ATP is released by microglia under hypotonic stimulation is based on severeal lines of evidence. First, we demonstrated purine release by ultraperformance liquid chromatography in cultured microglia. However, it is unlikely to observe ATP or ADP, which have been shown to be very unstable^55^ and the accumulation of adenosine represents a highly likely proof of their release^50^. In addition, we directly measured ATP efflux induced by cell swelling with a FRET based cytoplasmic ATP-sensor^56^. Moreover, we obtained an independent proof of ATP release in slices, using the paradigm of ATP-induced microglia processes recruitment^23, 31^. Indeed, our results show that the activation of I_Swell_ in hypotonic conditions is associated to ATP dose dependent recruitment of microglia processes. Although indirect, this assay has the advantage to highlight the functional effect of ATP release in the same conditions used for swelling activated currents. However, the nature of the channels or transporters involved in swelling-induced ATP release is unknown. Pannexin hemichannels mediate ATP release in astrocytes^57^ and neurons^17^. Moreover these channels are mechanosensitive^58^ and allow ATP release upon swelling^17^. Data from BV-2 cells support the involvement of pannexin hemichannels in microglia ATP release under hypotonic stimulation. Indeed, the decrease in intracellular ATP concentration is abolished both by CBX and probenecid, supporting pannexin mediated ATP release. This is consistent with results concerning pannexin involvement in I_Swell_ activation, which is prevented by both blockers in our conditions. On the other hand, gadolinium, a blocker of maxi anion channels, had no effect on I_Swell_. Based on these results, we hypothesized that in response to hypotonic stimulus, ATP or ADP, could leak from microglia cell through pannexins^59, 60^ (Fig. 8). It should be noted that the interpretation of CBX data is limited by the unselectivity of this compound^61^. Indeed, CBX might block directly both volume-activated currents^61–63^ and connexin hemichannels^64^. However, the inhibition of I_Swell_ with the selective pannexin blocker probenecid supports the involvement of pannexins in the mechanism leading to I_Swell_ activation.

Another relevant point concerns the role of Ca^2+^ in I_Swell_ activation. Our data strongly support a Ca^2+^-dependent mechanism, as we demonstrated that hypotonic stimulation induces [Ca^2+^]_i_ increase and that I_Swell_ is inhibited by intracellular Ca^2+^ buffering. Regarding the source of calcium necessary for the activation of the current, we highlighted a role for intracellular Ca^2+^. Indeed, we observed that I_Swell_ is not significantly affected by removal of extracellular Ca^2+^, but shows a significant reduction as a result of intracellular stores depletion by thapsigargin. Consistently with pharmacological evidences on purinergic receptors, we can argue the involvement of P2Y receptors in I_Swell_ amplification through intracellular Ca^2+^ increase. However, we found some discrepancy between the amplitude reduction observed with thapsigargin and the block caused by 30 mM intracellular BAPTA. It is possible that the full block could arise from direct effects of BAPTA on I_Swell_, similar to those reported in other cell types. Indeed, Cl^−^ channels are described as highly sensitive to extracellular BAPTA^65^, although in a dose range and experimental conditions very different from those here reported; in addition, a subpopulation of Cl^−^ channels in brown adipocytes is blocked by intracellular BAPTA^66^. On the other hand, reasoning that the action of high BAPTA is genuinely due to its Ca^2+^ chelating capacity, the strict dependency of I_Swell_ activation on Ca^2+^ buffering would suggest the involvement of a high affinity Ca^2+^-binding site in the mechanism of channel activation^67^. It should be noted that, increasing free intracellular Ca^2+^ was not sufficient to induce I_Swell_ activation, in the absence of the hypotonic stimulus. Thus, Ca^2+^ increase may represent a permissive factor, favoring current activation^16, 43^, rather by modulating volume-sensitive anion channels^16, 36, 68^, than by activating calcium-sensitive ones^16, 69, 70^. In addition, Ca^2+^ rise could amplify I_Swell_ activation by favoring pannexin-mediated ATP release^71, 72^.

Altogether, our data demonstrate that swelling-activated chloride current in native microglia cells is dependent on intracellular Ca^2+^ and associated to release of purines, likely causing current amplification by an autocrine pathway (Fig. 8).

Furthermore, our study raises the possibility that membrane swell and the consequent ATP release are used by microglia cells to send signals to other cells, providing a novel mechanism by which microglia could communicate and potentially affect neuronal activity.

## Methods

### Patch clamp recordings on slice

Procedures using laboratory animals were in accordance with the Italian and European guidelines and were approved by the Italian Ministry of Health in accordance with the guidelines on the ethical use of animals from the European Communities Council Directive of September 20, 2010 (2010/63/UE). All efforts were made to minimize suffering and number of animals used. Slices were prepared from 2–3 week old *Cx3cr1* ^+/*GFP*^ mice^73^. Animals were decapitated under halothane anesthesia. Brains were rapidly immersed in chilled oxygenated (95% O_2_, 5% CO_2_) artificial cerebrospinal fluid (ACSF) containing (mM): NaCl 125, KCl 2.5, CaCl_2_ 2, MgCl_2_ 1, NaHPO_4_ 1, NaHCO_3_ 26, glucose 10 (300 mOsm, micro-osmometer Roebling, type 13/13DR, Autocal). Transverse 250 μm hippocampal slices were cut (4 °C; vibratome DSK, Kyoto, Japan) and allowed to recover in oxygenated ACSF for at least 1 hour (room temperature, RT). GFP-expressing microglia were recorded in whole-cell configuration in CA1 stratum radiatum under continuous perfusion (1.5 ml/min). Micropipettes (4–5 MΩ) were filled with standard solution (mM): KCl 140, EGTA 0.5, MgCl_2_ 2, HEPES 10, Mg-ATP 2 (pH 7.3 with KOH, 290 mOsm). To change Ca^2+^ buffering, EGTA was substituted with BAPTA 0.5 mM without altering KCl concentration; when BAPTA was 5 or 30 mM, KCl was reduced to 135 mM or 95 mM, respectively. Ca^2+^ store depletion was obtained adding thapsigargin (1 μM) in the pipette solution. To determine Cl^−^ selectivity, we used a low Cl-intracellular solution, containing (mM): K-gluconate 140, EGTA 0.5, MgCl_2_ 2, HEPES 10, Mg-ATP 2 (pH 7.3 with KOH, 290 mOsm). N-Methyl-D-glutamine (NMDG)-based solution contained (mM): NMDG 150, EGTA 0.5, MgCl_2_ 2, HEPES 10, Mg-ATP 2 (pH 7.3 with HCl, 285 mOsm). The desired [Ca^2+^]_i_ was obtained by adding, to the intracellular solution, amounts of CaCl_2_ calculated with the webmax software (www.stanford.edu/cpatton/webmaxc/webmaxcS.htm).

Voltage-clamp recordings were performed at RT using Axopatch 200 A amplifier (Molecular Devices). Currents were filtered at 2 kHz, digitized (10 kHz) and collected using Clampex 10 (Molecular Devices); off-line was performed using Clampfit 10 (Molecular Devices). Recordings were completed in 5–6 hours after slicing and performed at least 25 μm under slice surface, to avoid microglia activated during slicing.

Unless otherwise stated, I_Swell_ was evoked exposing slices chronically to hypoosmotic ACSF (8–10% dilution of standard ACSF; in order to establish 20 mOsm Δ between pipette and extracellular solutions). Slices were placed after cutting in hypoosmotic medium and perfused with it during recordings. In specific experiments, hypoosmotic stimulus was applied acutely after 9 minutes of recording.

Resting membrane potential and membrane capacitance were monitored along the experiments. The current/voltage (I/V) relationship were determined applying voltage steps (50 ms) from −170 to +70 mV (HP = −70 mV; ΔV = 10 mV) every 3 minutes. I_Swell_ amplitude was measured by subtracting the current amplitude measured just after membrane rupture (chronic protocol; 12 min of recording) or that recorded just before hypotonic application (acute protocol; 18 min hypotonic stimulation). To generate I/V relationships, leak current was added to the recorded current amplitude at each time point. Although not quantified, cell swelling was observed during hypoosmotic challenge.

Data are presented as mean ± SEM; offline statistical analysis was performed with Origin 8 and SigmaPlot 11 (Systat Software Inc., San Jose, California, USA).

### Patch-clamp recording in cultured BV-2 cells

BV-2 cells were grown in Dulbecco’s Modified Eagle Medium with Glutamax (Gibco) supplemented with 10% heat-inactivated fetal bovine serum (FBS; Gibco), 100 U/ml penicillin G and 100 μg/ml streptomycin (Gibco) at 37 °C in a 5% CO_2_ humidified atmosphere. Cells were plated at density of 1–4*10^4^ cells in poly-L-lysine-coated glass coverslip (12 mm diameter) and used after 2–3 days.

Cells were patched using micropipette (4–5 MΩ) filled with standard intracellular solution described above. During the experiment cells were perfused with a normal extracellular solution (NES), containing (mM): NaCl 140, KCl 4; CaCl_2_ 2; MgCl_2_ 1; HEPES 10 (pH 7.34 with NaOH, 287 mOsm). Steps protocol and low chloride solution as in slices. Hypotonic stimulus (91 instead 140 mM of NaCl; 205 mOsm) was applied acutely after 4 minutes of recording. Data analyzed and presented as in slices.

### Primary murine microglia cultures

Microglia cultures were prepared from postnatal day 0–1 C57bl6 mice. Briefly, cortices were chopped and digested in 15 U/ml papain (20 min; 37 °C). Cells (5 × 10^5^ cells/cm^2^) were plated on flasks poly-L-lysine coated flasks (100 μg/ml) in DMEM supplemented with 10% FBS, 100 U/ml penicillin and 0.1 mg/ml streptomycin. After 9–11 days, cultures were shaken (2 h; 37 °C), giving almost pure microglia cell populations. For the experiments, cells were plated on 12 mm glass poly-L-lysine coated coverslips (5 × 10^4^ cells/coverslip).

### Cl^−^-Imaging in cultured BV-2 cells

BV-2 cells were transfected using magnetotrasfection technique (OZ Bioscience) with cDNA of Cl-Sensor^24, 25, 74^.

Fluorescence signals were acquired as in Bertollini *et al*.^25^. Briefly, BV-2 cells expressing Cl-Sensor were visualized with an upright microscope (Axioskope) with 40x water-immersion objective (Achroplan CarlZeiss, USA) and a digital 12 bit CCD camera (SensiCam, PCO AG, Germany) and excited with a momchromator (Till Polychrome V) with a 150 W xenon lamp (Till Photonics, Germany) alternatively at 445 nm and 485 nm wavelength (50 msec, 0.1 Hz); [Cl^−^]_i_ changes are expressed as ratio of background subtracted F_445_ over F_485_ (F_445_/F_485_) (Supplementary Fig. S3). Peripheral hardware control, image acquisition and processing were achieved using customized software Till Vision v. 4.0 (Till Photonics); Origin 6.0 was used for offline analysis.

To induce cell depolarization and increase driving force for chloride, NES was replaced with a high K^+^ solution (High K^+^), containing (mM): NaCl 84, KCl 60, CaCl_2_ 2, MgCl_2_ 1, HEPES 10 (pH 7.34 with NaOH, 287 mOsm). Hypotonic stimulus was applied using a modified high K^+^ solution (Hypotonic High K^+^: 34 instead 84 mM NaCl; 205 mOsm).

### Tracking analysis of single microglia processes

Microglia processes tracking was performed as previously described^23^. Briefly, images were processed using ImageJ software and data analyzed with ImageJ and Origin 8 (OriginLab Co.) software to obtain quantitative distributions of track parameters The ability of the processes to be attracted towards the pipette tip with different ATP-concentration was defined as ΔR = R_f_ − R_0_, where R_0_ and R_f_ are the distances from the initial and last sample point of the track i respectively. Negative ΔR values reflect directed process movement to the pipette tip. ATP-dependent displacement was expressed as a function of radial distances of microglia processes from the pipette tip in normotonic and hypotonic medium conditions.

### FRET analysis of cytosolic ATP concentration

To monitor single-cell cytosolic [ATP] changes, primary microglia and BV-2 mouse microglia cells were transfected with a plasmid encoding the ATP sensor AT1.03^59^, using 2 μl Lipofectamine-2000 and 1 μg DNA per 250 μl of medium (50 μl OptiMem and 200 μl of the cell transfection medium). Incubation medium was removed leaving 200 μl per well and 50 μl of DNA/Lipofectamin mixure was added into each well. Cells were maintained in CO_2 _incubator (37 °C, 2–2.5 h), then medium was discarded and cells washed twice with fresh medium. Fluorescence measurements were performed 24 hours after transfection^59^ using inverted microscope Olympus IX73 (Olympus Europe) with a 40× objective. Fluorescence of AT1.03 was excited at 436 nm with a xenon lamp (Lumen 200PRO, Prior) using a filter wheel (X-light, CrestOptics); emission was monitored at 527 nm and 475 nm using an emission splitting system (DV2, Photometrics). Images were acquired by cooled CCD camera (CoolSNAP Myo, Photometrics). Imaging data were collected and analyzed using MetaFluor 6.1 (Molecular Device, USA). Cells were treated with CBX (100 μM), probenecid (500 μM and 1 mM) or FFA (200 μM) for 30 minutes before FRET experiments and during the entire time lapse.

### Ultra-performance/pressure Liquid Chromatography

Samples were collected from multiwell plated primary microglia cultures (1*10^5^ cells/well); after 24 hours after plating, the culture medium was removed and cells were equilibrated for 16 hours in NES-340 (NES adjusted to 340 mOsm by adding NaCl), isotonic to the culture medium. Cells were, then, exposed to hypotonic stimulus, by diluting NES-340 to 255 mOsm with water. An equal volume of NES-340 was added to control microglia cell, in order to treat cells similarly, maintaining osmolarity unaltered. From each sample, the total volume was collected and centrifuged at 14000 g for 30 minutes at 4 °C and 380 μl of supernatants were immediately stored at −80 °C. Samples were then concentrated by freeze-drying, suspended in 50 μl of ultrapure water, centrifuged at 14000 g (30 minutes, 4 °C) and 10 μl of supernatant were injected directly onto UPLC column. Chromatographic determination and quantification of ATP and adenosine nucleotides was performed on a Waters Acquity H-Class UPLC system (Waters, Milford, MA, USA), including quaternary solvent manager (QSM), sample manager with flow through needle system (FTN), and photodiode array detector (DAD). Column temperature, 30 °C; flow rate 0.3 ml/min; peaks detected at 260 nm. The method employs a reverse-phase column with the use of phosphate buffer in mobile phase to enhance retention and separation of the compounds of interest^75, 76^.

### Statistical analysis

Paired and unpaired t-test, one-way and two-way ANOVA were used for parametrical data; for multiple comparisons (Holm-Sidak method), multiplicity-adjusted p values are indicated in figures when appropriate.

### Drugs

Drugs and reagents used were purchased from Sigma Aldrich, Life Technologies, Ascent Scientific and Invitrogen.

## Electronic supplementary material

Supplementary Figures
