# Supplementary material for: ATP release during cell swelling activates a Ca2+-dependent Cl− current by autocrine mechanism in mouse hippocampal microglia
Source: Sci Rep. 2017 Jun 23;7:4184. doi: 10.1038/s41598-017-04452-8 (PMC5482828; doi:10.1038/s41598-017-04452-8)
Supplement: Supplementary file 1 — Supplementary Figures [file 41598_2017_4452_MOESM1_ESM.pdf]

ATP release during cell swelling activates a Ca^{2+}-dependent Cl^- current by autocrine mechanism in mouse hippocampal microglia

Murana, E., Pagani, F., Basilico, B., Sundukova, M., Batti, L., Di Angelantonio, S., Cortese, B., Grimaldi, A., Francioso, A., Heppenstall, P., Bregestovski, P., Limatola, C., Ragozzino, D.

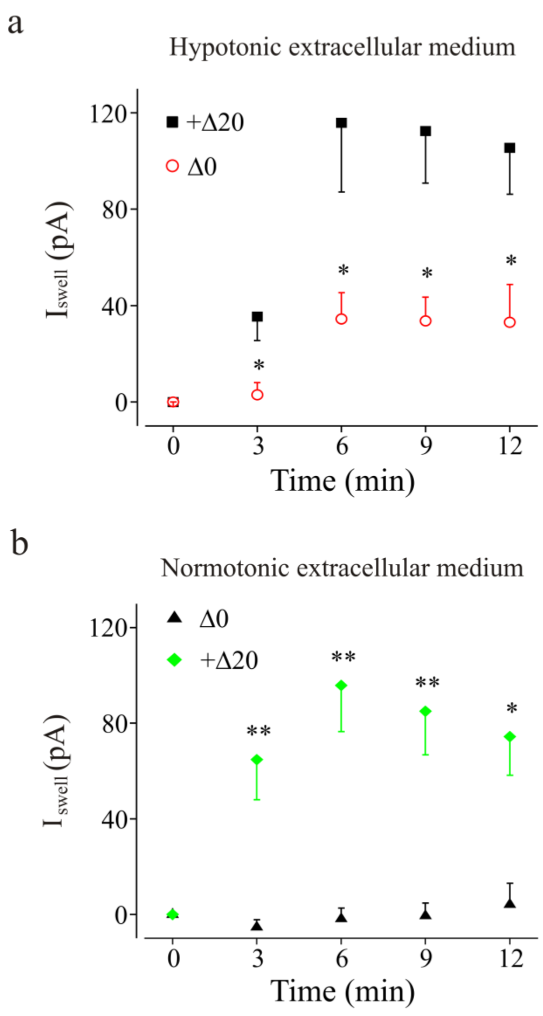

Supplementary Figure 1-Murana et al.

Supplementary figure 1.

a. Time-course of I_{swell} recorded in microglial cells in hypotonic medium. Pipette solutions were manipulated in order to obtain different osmolarity deltas. When intracellular osmolarity was higher (Δ20 mOsm; +Δ20, n = 22; black) I_{swell} was typically activated. This was not the case in the absence of osmolarity delta (Δ0, n = 16; red; * $p < 0.05$, t-test).

b. Time-course of I_{swell} activation as in **a**, in normotonic extracellular solution in the absence of osmolarity delta (Δ0, n = 11; black) or with delta osmolarity of 20 mOsm (+Δ20, n = 13; green; **, $p < 0.01$, t-test).

Notice that, the full activation of I_{swell} was achieved only in the presence of an osmotic delta across microglia cell membrane, both in normotonic or hypotonic medium.

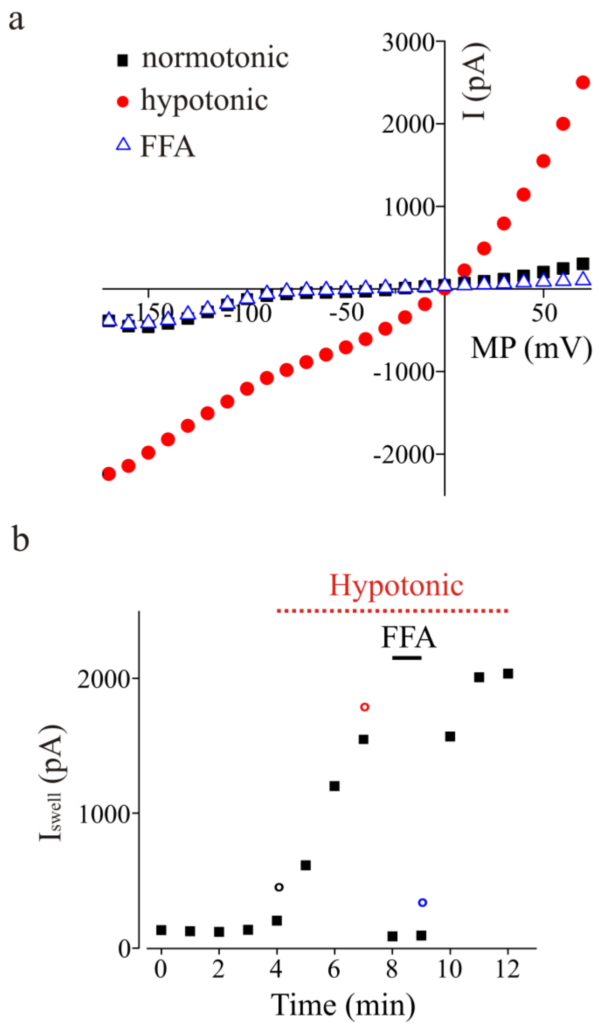

Supplementary Figure 2-Murana et al.

Supplementary figure 2.

a. Representative current-voltage relationships in a BV-2 cell during hypotonic stimulation. Control (black), hypotonic medium (205 mOsm; red), FFA application (200 µM; blue) as indicated by dots in b.

b. Inhibition by flufenamic acid (200 µM) of the swell-activated current recorded in a BV-2 cell under acute hypotonic stimulation.

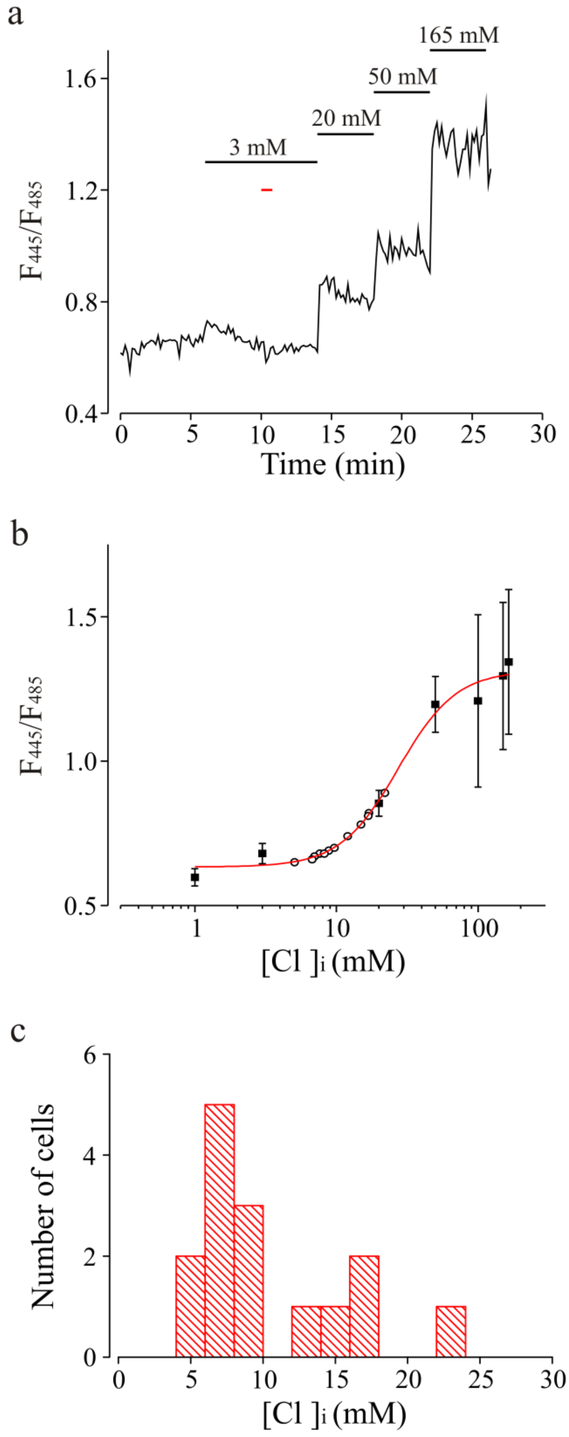

Supplementary Figure 3-Murana et al.

Supplementary figure 3.

a. Representative calibration performed in Cl-Sensor BV-2 transfected cells. Red line: β-escin, 80 μM, 1 min; black lines: application of extracellular solutions with the indicated concentration of Cl^-. Calibration of Cl-Sensor expressed in BV-2 transfected cells was performed as in Wassem et al. External solutions with different Cl^- concentrations (in mM: 1, 3, 20, 50, 100, 150, 165) were obtained by mixing the following solutions (in mM): (i) 164.8 KCl, 10 D-glucose, 20 HEPES, pH 7.32 and (ii) 164.8 K-gluconate, 10 D glucose, 20 HEPES, pH 7.32. To increase the permeability of the cell membrane to Cl^- ions, β-escin (80 μM, Sigma, St Louis, MO, USA) was added to the extracellular solution and applied for 1 min. Fluorescence signals, corresponding to rising in intracellular Cl- concentrations, were monitored from single Cl-Sensor expressing BV-2 cell, after β-escin removal.

b. Calibration curve of intracellular chloride in BV-2 cells transfected with Cl-Sensor. Each black square represents the mean (± SEM) fluorescence ratio measured at different chloride concentration (n = 5-14 cells). Data were best fitted using the logistic equation: $y=((A_1-A_2))/(1+(x/x_0)^p)+A_2$

Where:

$A_1=R_{min}$, $A_2= R_{max}$, $x_0=[Cl^-]$ at $y=R_{max}/2$, p= nH. Notice that R_{max} was estimated at maximal $[Cl^-]$, 165 mM.

White circles refer to the fluorescence ratio values in basal condition, evaluated in BV-2 cells transfected with Cl-Sensor, fitted on the calibration curve (n =15).

c. Distribution of basal $[Cl^-]_i$ estimated for BV-2 cells.

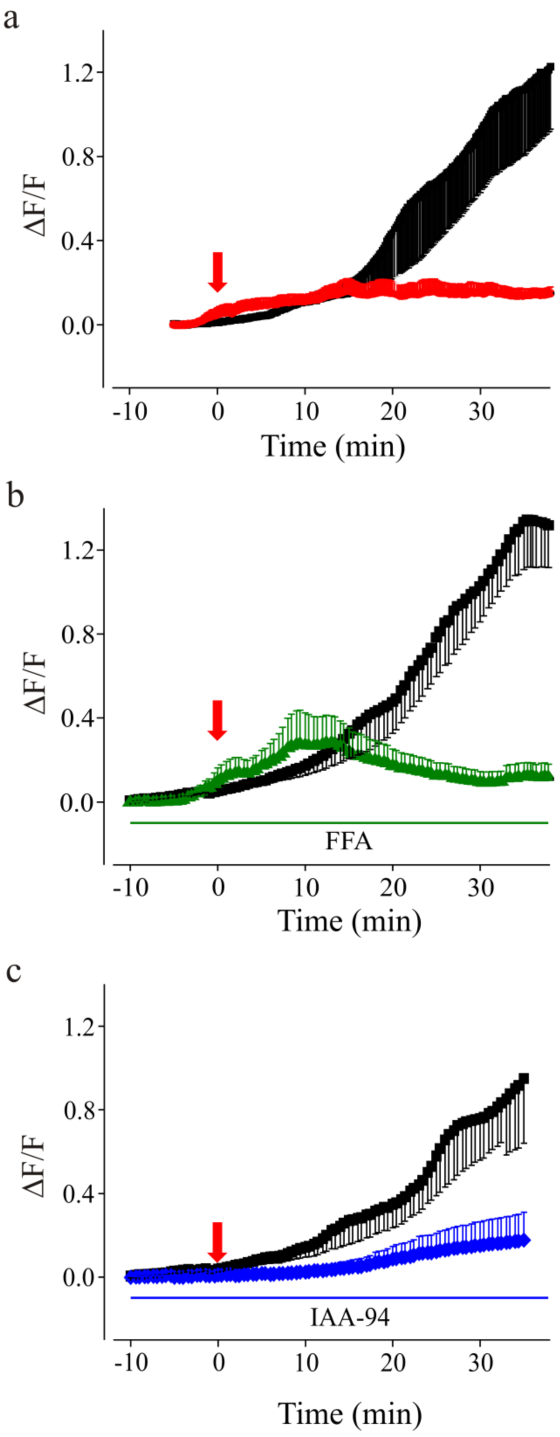

Supplementary Figure 5-Murana et al.

Supplementary figure 4.

A glass pipette containing adenosine 5'-triphosphate magnesium salt (ATP, 2 mM; Sigma Aldrich) was placed in the stratum radiatum of CA1 hippocampal Cx3cr1-GFP mouse slice. Mg-ATP was pressure applied to the slices (100 ms; 5 psi) with a Picospritzer III (Parker Instrumentation) as in Pagani et al., (2015). Changes in GFP fluorescence distribution were monitored by acquiring a

fluorescent image every 10 seconds for 50 minutes. To quantify the speed of microglial processes rearrangement toward the pipette tip, we measured the increase of GFP fluorescence in concentric areas centered on the pipette tip (10, 20, 50, 80 µm radius). At each time point the fluorescence increase in the areas was calculated as $F = F-F_0$, and then normalized for F_0 (where F_0 is the average fluorescence before ATP puff). Slices were used from 2 to 6 hours after cutting.

a. Time course of fluorescence (F/F_0) measured in the 10 µm radius circle centered on the tip of the ATP puff pipette (arrow indicates ATP application) in slices incubated in control condition (black; n = 9) and in a Na-gluconate based ACSF (red; n = 6).

b. Time course of F/F_0, measured as in a, in control condition (black; n = 9) and in slices treated with flufenamic acid (FFA, 200 µM, green symbols; n = 6). Arrow as in A.

c. Time course of F/F_0, measured as in A, B, in control condition (black; n = 8) and in the presence of indanyloxyacetic acid 94 (IAA-94, 1 mM, blue; n = 5). Arrow as in a.

Notice that both Cl^- removal and anionic channel block reduce ATP-induced microglial processes rearrangement.
